# Flowering of Woody Bamboo in Tissue Culture Systems

**DOI:** 10.3389/fpls.2017.01589

**Published:** 2017-09-14

**Authors:** Jin-Ling Yuan, Jin-Jun Yue, Xiao-Ping Gu, Choun-Sea Lin

**Affiliations:** ^1^Research Institute of Subtropical Forestry, Chinese Academy of Forestry Hangzhou, China; ^2^Agricultural Biotechnology Research Center, Academia Sinica Taipei, Taiwan

**Keywords:** flowering induction, *in vitro* seed set, *in vitro* hybridization, bamboo reproduction, plant growth regulators

## Abstract

Flowering and subsequent seed set are not only normal activities in the life of most plants, but constitute the very reason for their existence. Woody bamboos can take a long time to flower, even over 100 years. This makes it difficult to breed bamboo, since flowering time cannot be predicted and passing through each generation takes too long. Another unique characteristic of woody bamboo is that a bamboo stand will often flower synchronously, both disrupting the supply chain within the bamboo industry and affecting local ecology. Therefore, an understanding of the mechanism that initiates bamboo flowering is important not only for biology research, but also for the bamboo industry. Induction of flowering *in vitro* is an effective way to both shorten the flowering period and control the flowering time, and has been shown for several species of bamboo. The use of controlled tissue culture systems allows investigation into the mechanism of bamboo flowering and facilitates selective breeding. Here, after a brief introduction of flowering in bamboo, we review the research on *in vitro* flowering of bamboo, including our current understanding of the effects of plant growth regulators and medium components on flower induction and how *in vitro* bamboo flowers can be used in research.

## Introduction

Flowering, fruiting, and seed development are the most fundamental processes of sexual propagation in plants. Most flowering plants pass from seed germination to a brief period as a seedling, to a vegetative or juvenile phase that is predominated by growth, and then onto a reproductive phase, during which plants have the capacity to produce the components required for flowering and seed production (Huijser and Schmid, 2011). The length of the plant juvenile phase varies widely. Usually, herbaceous plants have a short juvenile phase (within 1–2 seasons), complete their life cycle within a few years, and die after seed production (Feng et al., 2016). However, woody plants have a long juvenile phase (many years), remain alive after flowering, and can flower every year after reaching maturity (Wendling et al., 2014a,b).

**Figure 1:**
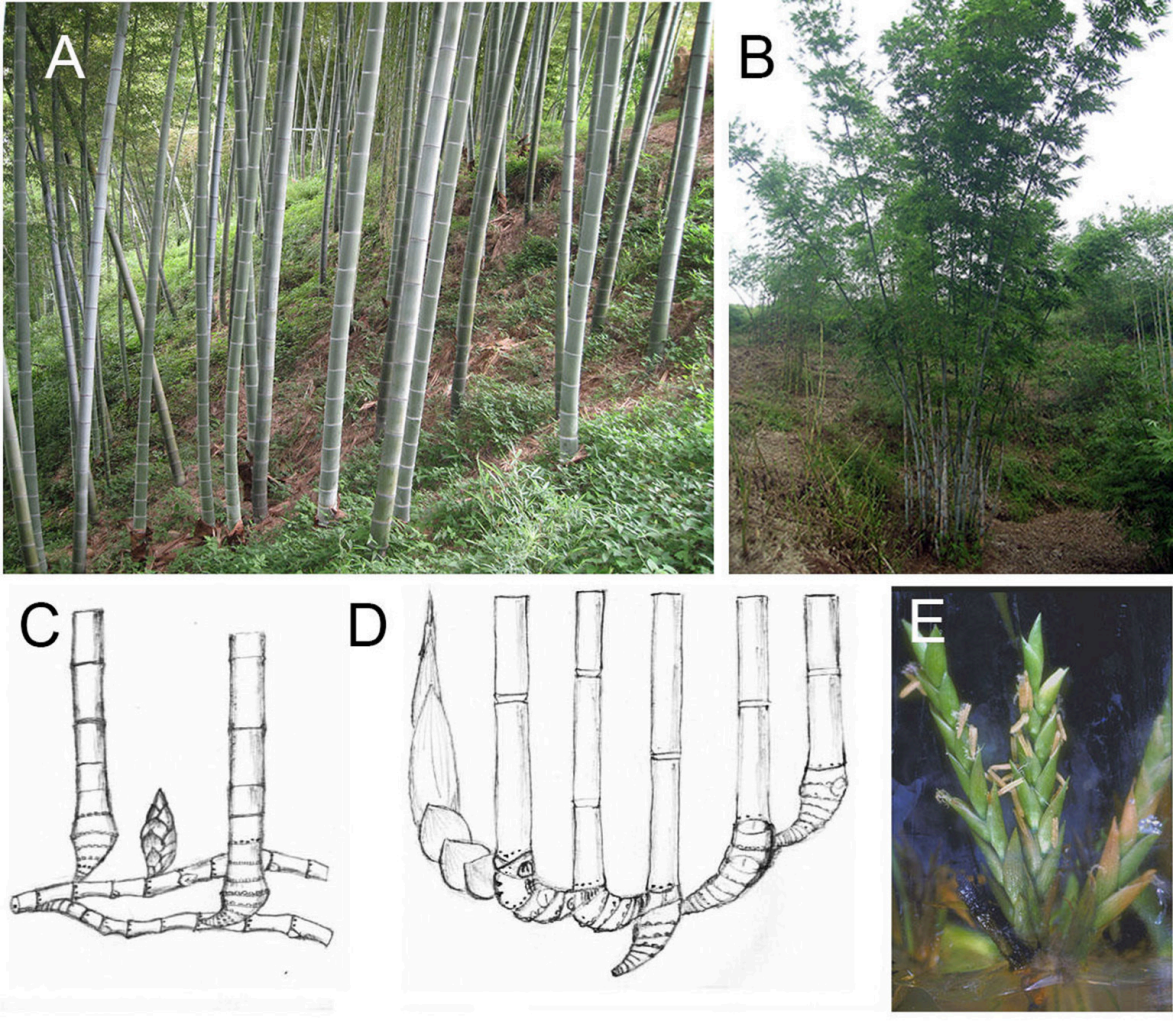
Stalks, rhizomes, and flower of monopodial and sympodial bamboo. **(A)** Stalks of a monopodial bamboo (*P. edulis*). **(B)** Stalks of a sympodial bamboo (*B. chungii*). **(C)** Rhizome of monopodial bamboo. **(D)** Rhizome of sympodial bamboo. **(E)** Flower in tissue culture system of a sympodial bamboo (*B. edulis*).

Compared with these two types of plants, woody bamboos which were identified as monopodial with leptomorph rhizome (Figures 1A,C) and sympodial with pachymorph rhizome (Figures 1B,D; McClure, 1966), have a unique flowering behavior. Woody bamboos have a very long juvenile phase (decades), similar to woody plants. However, woody bamboo only flowers once and dies after seed production (monocarpy) (McClure, 1966; Janzen, 1976).

There are advantages and disadvantages to this unique flowering behavior, particularly for the bamboo industry. During the long vegetative phase, bamboo stalks (building materials) and young shoots (edible vegetable) can be continually harvested for many years. Propagation costs can be reduced in bamboo species that grow by rhizomes, such as monopodial bamboos, yielding an entire plantation composed of plants of the same genetic background. These monoculture plantations consist of plants initiated at the same time and often flower en masse, disrupting the supply chain and causing huge economic losses. This gregarious flowering not only takes that plantation out of service (Sarma et al., 2010) but also causes ecological and enviromental challenges. For example, a bamboo grove that has recently flowered does not provide food for the giant panda (Li and Denich, 2004). Furthermore, once fruit has set, this new food source can lead to overpopulation of rats, which in the past have over-consumed the seeds, leaving the bamboo forest unrecovered (Nag, 1999).

Since it is difficult to predict flowering time and to time the flowering of two bamboo accessions for hybridization, it is difficult for breeders to use select varieties for bamboo breeding (John and Nadgauda, 1999; Singh et al., 2013). Without genetic recombination through cross-pollination, genome diversity is limited and genetic studies are nearly impossible. Furthermore, bamboo classification is debated and confusing since plant taxonomy often relies on the morphology and anatomy of flowers and fruits and speciation depends on sexual incompatibility (Bhattacharya et al., 2006, 2009).

Over the years, many researchers have tried to manipulate bamboo flowering not only for research and industrial purposes but also to manage the environmental impact. Because of the size of woody bamboo, it is difficult to establish controlled environments for scientific research. The first case of *in vitro* bamboo flowering and seed production was reported less than 30 years ago (Nadgauda et al., 1990). Compared with *in vivo* flowering, there are many advantages to *in vitro* flowering. Firstly, the plantlets can be incubated in a sterile, controlled environment, which can reduce interference from biotic and abiotic stresses and uncontrolled pollination. Secondly, the size of the plantlet is relatively small within the incubation container, allowing addition of plant growth regulators to the whole plantlet. Thirdly, flowering can be induced when desired. Here, we review recent reports on *in vitro* bamboo flowering.

## Flower induction *In vitro*: species and explant types

To date, 13 bamboo species have been induced to flower *in vitro* (Table 1). Five of the species belong to the genus *Bambusa*: *B. arundinacea* (Nadgauda et al., 1990, 1997; Ansari et al., 1996; Joshi and Nadgauda, 1997), *B. edulis* (Lin and Chang, 1998, Figure 1E), *B. multiplex* (Prutpongse and Gavinlertvatana, 1992), *B. oldhamii* (Ho and Chang, 1998; Zhang and Wang, 2001), and *B. vulgaris* (Rout and Das, 1994). Six of the species belong to the genus *Dendrocalamus*: *D*. *brandisii* (Nadgauda et al., 1990), *D. giganteus* (Rout and Das, 1994; Ramanayake et al., 2001), *D. hamiltonii* (Chambers et al., 1991; Kaur et al., 2015), *D. latiflorus* (Zhang and Wang, 2001; Lin et al., 2006, 2007b), *D. membranaceus* (Prutpongse and Gavinlertvatana, 1992), and *D. strictus* (Rout and Das, 1994). The last two accessions are *Cephalostachyum pergracile* (Prutpongse and Gavinlertvatana, 1992) and an intergenus hybrid, *B. pervariabilis* × *D. latiflorus* (Zhang and Wang, 2001). Based on these reports, all the bamboo species that have been shown to flower *in vitro* are sympodial bamboos. There has not been a report on *in vitro* flowering for a monopodial bamboo. Actually, there are only few reports on monopodial bamboo tissue culture (Gielis, 1999; Wang et al., 2005; Pei et al., 2011; Mudoi et al., 2013; Yuan et al., 2013), regardless of its better cold-tolerance and other merits over sympodials. Although Hassan and Debergh (1987) originally reported tissue culture protocols for *P. viridis*, they retracted the article because of a taxonomy issue. Therefore, work remains to be done to develop *in vitro* flowering tissue culture protocols for important monopodial species.

**Table 1:** *In vitro* flowering of bamboo species: explant types, medium components, and results.

**Species**	**Explant**	**Medium**	**Induction time**	**Main results**	**References**
*Bambusa arundinacea, Dendrocalamus brandisii, D. strictus*	Seedling	MS + 2% sucrose + 5 mg/L BA + 50 ml/L CW		70% *B. arundinacea* and 40% *D. brandisii* flowered, and fertile seeds produced.	Nadgauda et al., 1990
*B. arundinacea*	Seedling	MS + 3% sucrose + 0.7% agar + 2 mg/L BA + 3 mg/L NAA	Rooting at 10 days. Flowering at 45 days.	Peroxidase depressed prior to appearance of root and flower primordia.	Ansari et al., 1996
*B. arundinacea*	Seedling	MS + 2% Sucrose + 5% CW + 2.2 μM BA	3–6 months	About 70% of cultures flowered, pollen fertility approximately 31%, *in vitro* seeds were produced.	Nadgauda et al., 1997
*B. arundinacea*	Seedling	MS + 2% sucrose + 2.22 μM BA, or with 0.26 μM ZT, 2.71–271.0 μM AdS, 2.32–9.30 μM Kin, 4.9 μM 2iP		*In vitro* florets had all floral parts. BA was the only cytokinin to induce flowering, either individually or with others (ZT, AdS, Kin, 2iP). Root elongation and flower induction had an inverse relationship.	Joshi and Nadgauda, 1997
*B. arundinacea, B. multiplex, Cephalostachyum pergracile, D. brandisii, D. membranaceus*	Nodal explants obtained from field	MS + 22.2 μM BA		Only *B. multiplex* survived after flowering.	Prutpongse and Gavinlertvatana, 1992
*B. edulis*	10-year-old field-grown nodal explants	MS + 0.1 mg/L TDZ	8 months	Spikelets and florets normal, no seed set, no viable pollen produced. A potted plant flowered and survived after flowering.	Lin and Chang, 1998
*B. edulis*	Inflorescence	MS + 0.1 mg/L TDZ + 30 g/L sucrose	10 months, subcultured every 21 days	Inflorescence proliferated, pollen sterile.	Lin C. S. et al., 2003
*B. edulis*	*In vitro* spikelet	MS + 0.5 μM TDZ, or 23.2 μM Kinetin, or 16.2 μM BPA, or 22.2 μM BA, or 22.8 μM ZT, + 30 g/L sucrose	21 days	Cytokinins were effective in flower induction, but NAA was a negative regulator. Rooted plantlets with vegetative shoots, even though they had flowers, could survive and grow well as normal plants without hardening after transplant to greenhouse.	Lin C. C. et al., 2003
*B. edulis*	Shoots from somatic embryo-derived plants	MS + 0.455 μM TDZ		Flowers had anthers with pollen grains, but sterile pollen. Plantlet flowering *in vitro* survived after transferring into greenhouse.	Lin et al., 2004a
*B. edulis*	Inflorescence	MS + 0.1 mg/L TDZ	Long-term proliferation	TDZ, but not GA_3_, ABA, or ACC alone, was efficient in inducing inflorescence proliferation.	Lin et al., 2004b
*B. edulis*	Inflorescence	MS + 5 mg/L NAA; MS + 5 mg/L NAA + 1 mg/L ACC	2 months	NAA, IBA, 2, 4-D induced vegetative shoots. 50% of shoots flowered in MS + 5 mg/L NAA, and all flowered in MS + 5 mg/L NAA + 1 mg/L ACC. All rooted plantlets survived after transplanting in greenhouse.	Lin et al., 2005
*B. oldhamii*	Eleven- year old embryogenic cell line	MS + 3 mg/L 2,4-D + 2 KT + 6% sucrose		Five percent albino plantlets flowered and produced viable pollen. Seventy five percent of the pollen was fertile.	Ho and Chang, 1998
*B. pervariabilis × D. latiflorus, D. latiflorus*	Seedling of *D. latiflorus*, nodal explants of *B. pervariabilis × D. latiflorus*	3/4MS + 2–4 mg/L BA +0.5–1.0 mg/L KT + 100 ml/L CW	1–3 years	Seedling clones of *D. latiflorus* with strong shoot-emerging ability were easily induced to flower. BA was effective in inducing flowering. KT was helpful for vegetative growth.	Zhang and Wang, 2001
*B. vulgaris, D. giganteus, D. strictus*	Nodal explants from somatic embryo-derived plants	MS + 0.5 mg/L Ads + 0.25 mg/L IBA + 0.5 mg/L GA_3_ + 3% sucrose	12 weeks	*In vitro* flowering was achieved. About 10–12 viable seeds were obtained from each culture of *D. strictus* and *D. Giganteus*; *B. vulgaris* produced 3-4 seeds per culture.	Rout and Das, 1994
*D. giganteus*	Adult bamboo node segment	MS + 2% sucrose + 3–6.0 mg/L BA + 0.1 mg/L KT	29 months	The lemma tapered to a point and the margins opened out. Spikelets were narrow and long. Stamens ranged from 0 to 12. Anthers did not dehisce. Microspores were empty. Neither the style nor the stamens elongated as in the field. No seed set.	Ramanayake et al., 2001
*D. hamiltonii*	Seedling	MS + 2% sucrose + 5 mg/L BA + 50 ml/L CW, MS + 4.4–44 μM BA	13–15 weeks	The stigmas exited firstly from the palea and lemma, followed several days later by stamens. Pollens viable. No fertilization or seed set.	Chambers et al., 1991
*D. hamiltonii*	*In vitro* shoots from somatic embryo derived plants	MS + 2% sucrose + 0.5 mg/L BAP + 0.25 mg/L IBA	14–35 days	Flowering induced from 27–80% of shoots at 14–35 days. A marked reduction in leaf size/area during flowering.	Kaur et al., 2014, 2015
*D. latiflorus*	Albino inflorescence	MS + 0.45 μM TDZ + 30 g/L sucrose	Long-term proliferation with 21-days subculture	TDZ induced long-term inflorescence proliferation, while TDZ combined with NAA inhibited it. NAA induced root and then shoot. 2,4-D, picloram induced shoot.	Lin et al., 2006
*D. latiflorus*	Inflorescences	MS + 0.1-1.0 mg/L TDZ; MS + 1 mg/L BA; MS + 1 mg/L ZT	21 days to 8 months	Flower organs normal; pollen sterile.	Lin et al., 2007b
*D. strictus*	Seedling	1/2MS + 2% sucrose + 0.5–1.0 mg/L TDZ	2 months	Anthers protruded only partially. Gynoecium remained within. Anthers failed to dehisce. About 20% of anthers were normal and 80% empty. No seed set.	Singh et al., 2000

*MS, Murashige and Skoog medium; 2,4-D, 2,4-dichlorophenoxyacetic acid; BPA, N-Benzyl-9-(2-tetrahydropyranyl) adenine; CW, coconut water*.

Different species showed different responses in the same medium. In medium supplemented with 6-benzylaminopurine (BA) and coconut milk, *B. arundinacea* showed a 70% flowering rate, *D. brandisii* only 40%, and *D. strictus* did not flower (Nadgauda et al., 1990). *D. brandisii* and *Dendrocalamopsis oldhamii* (=*B. oldhamii*) did not flower in a medium that could induce flowering in *B. pervariabilis* × *D. latiflorus* (Zhang and Wang, 2001). *B. edulis* flowered in a medium supplemented with 0.1 mg/L thidiazuron (TDZ), but *B. oldhamii* only proliferated multiple shoots (Lin and Chang, 1998; Lin et al., 2007a). Those reports indicated that different species will not induce flowering in a uniform medium, and the medium components for certain bamboo must be screened through purposely designed experiments.

The time to *in vitro* flowering also varies between different species, across a range including 45 days (*B. arundinacea*, Ansari et al., 1996), just under 12 months (*B. edulis*, Lin and Chang, 1998), 29 months (*D. giganteus*, Ramanayake et al., 2001), and three years (*D. latiflorus*, Zhang and Wang, 2001). Although it still takes years for some of the bamboos to flower, *in vitro* culture nevertheless dramatically reduces bamboo flowering times compared to those in the field.

Another key factor in *in vitro* flowering is the choice of explant used to establish the *in vitro* culture and the explant selected for subsequent micropropagation. Current protocols use shoot meristems (Lin and Chang, 1998; Ramanayake et al., 2001; Lin et al., 2010) and seedlings (Nadgauda et al., 1990, 1997; Chambers et al., 1991; Ansari et al., 1996; Joshi and Nadgauda, 1997; Singh et al., 2000; Zhang and Wang, 2001). However, sourcing of seeds is not predictable, and bamboo seeds often do not have unique or widely diverse genetic backgrounds. The use of meristems from superior bamboo lines is a better strategy that will support bamboo breeding.

## Flower induction *In vitro*: control by plant growth regulators

Plant growth regulators are critical to *in vitro* bamboo flowering. *In vitro* flowering of bamboo can be induced by cytokinins, as has been shown in *D. brandisii* (Nadgauda et al., 1990), *D. giganteus* (Rout and Das, 1994; Ramanayake et al., 2001), *D. hamiltonii* (Chambers et al., 1991), *D. latiflorus* (Zhang and Wang, 2001; Lin et al., 2007b), *D. strictus* (Rout and Das, 1994; Singh et al., 2000), *B. arundinacea* (Nadgauda et al., 1990, 1997; Joshi and Nadgauda, 1997), *B. edulis* (Lin and Chang, 1998; Lin C. C. et al., 2003), *B. multiplex* (Prutpongse and Gavinlertvatana, 1992), and *B. vulgaris* (Rout and Das, 1994, Table 1). The effects of cytokinins on *in vitro* bamboo flowering are species dependent. For example, kinetin (Kin) could not induce flowering in *B. arundinacea* (Joshi and Nadgauda, 1997) or *D. latiflorus* (Zhang and Wang, 2001), but could for *B. edulis* plantlets with multiple shoots (Lin C. C. et al., 2003). Similar positive results were observed with zeatin (ZT) treatment of *B. arundinacea* and *B. edulis* (Joshi and Nadgauda, 1997; Lin C. S. et al., 2003). In *B. arundinacea*, flowering only occurred in medium containing BA combined with either ZT, adenine sulfate (Ads), Kin, or isopentyl adenine (2iP), but not those containing only one of the listed cytokinins without BA (Joshi and Nadgauda, 1997). *D. strictus* could not flower with 5 mg/L BA alone (Nadgauda et al., 1990), but a combination of cytokinin (Ads), auxin [Indole-3-butyric acid (IBA)] and Gibberellic acid (GA_3_) could induce flowering and seed formation (Rout and Das, 1994). In *B. edulis*, cytokinins are important not only for flower induction but also for inflorescence proliferation (Lin C. S. et al., 2003). The inflorescences could multiply when treated with different kinds of cytokinins, such as BA (Lin et al., 2004b). According to these results, cytokinins play positive roles in bamboo flowering.

Interestingly, auxins play an opposite role in bamboo flowering. In medium containing 0.1 mg/L TDZ, flowering of *B. edulis* plantlets with multiple shoots was inhibited by naphthaleneacetic acid (NAA) (Lin C. S. et al., 2003). When using *in vitro* inflorescences as explants, auxin-only medium increased the floret size, and also induced adventitious roots and caused 35% more vegetative shoots to emerge. These rooted vegetative plantlets could be transplanted to the greenhouse and survive (Lin et al., 2005). These results indicated that auxin plays a negative role in bamboo flowering and inflorescence proliferation *in vitro*.

Other plant growth regulators and medium components have also been investigated, such as the ethylene precursor 1-amino-cycliopropane-1-carboxylic acid (ACC), acetic salicylic acid, gibberrellin, the gibberrellin synthesis inhibitor ancymidol (Lin, 1998), coconut water (Zhang and Wang, 2001), sucrose, nitrogen at various concentrations (Lin C. C. et al., 2003), and the pH of the medium (Joshi and Nadgauda, 1997). These treatments led to only slight effects on flower induction.

## Fertility of *In vitro*-induced flowers

Seeds could be obtained from *in vitro* flowers of *B. arundinacea, D. brandisii, B. vulgaris, D. giganteus*, and *D. strictus* (Nadgauda et al., 1990, 1997; Rout and Das, 1994). While *D. strictus* could produce fertile pollen grains (Singh et al., 2000), *in vitro* anthers of *B. edulis* could not (Lin and Chang, 1998; Lin C. S. et al., 2003; Lin et al., 2004a). In *B. edulis*, the effects of different plant growth regulators on fertility were analyzed. Although auxin treatments promoted anther emergeance outside of glumes, no fertile pollen or seeds were obtained (Lin et al., 2004b). During normal *in vivo* flowering, *D. strictus* and *B. multiplex* have good fertility and easily produce seeds (Nadgauda et al., 1993; Yuan et al., 2011), but there is no report of seed set in *Bambusa edulis*, reflective of the *in vitro* results. Therefore, we speculate that the differential fertility *in vitro* may be related to genetic characteristics of the bamboo species. There is evidence that *B. edulis* is an intergenus hybrid between *Bambusa* and *Dendrocalamus* (Ye, 2010; Zheng, 2014), meaning that *B. edulis* cannot produce gametes with the correct chromosome number for seed set. Due to its long juvenility, it is difficult to conduct cytogentics in bamboo using reproductive organs, such as anthers. Therefore, most karyotyping has been conducted using root tips (Chen R. Y., 2003), although these experiments may have resulted in unreliable chromosome counts in bamboo.

## Applications of bamboo *In vitro* flowering—cloning of flower-related genes

Bamboo flowers produced *in vitro* provide an important material for flower-related molecular and cell biology studies. *D. latiflorus* spikelets have been used to identify numerous full-length cDNAs of the flowering-related MADS genes (Chen Y. Y., 2003). From a *B. oldhamii* cDNA library, 4,470 (floral tissue) and 3,878 (vegetative tissue) ESTs were published (Lin et al., 2010). Using proteomic analysis of bamboo flowers, 128 differentially expressed proteins in floral meristems were identified (Kaur et al., 2015). To do such studies on gene and protein expression in floral organs, flowers must be readily available in sufficient quantity.

With next generation sequencing, it has become easier to investigate non-model plant transcriptomes. One such transcriptome that has been explored is that of the *in vitro*-produced flowers of *B. edulis*. Using this transcriptome and sequences from a bacterial artificial chromosome (BAC) library, 16 full-length Type II MADS (*BeMADS*) genes were identified. The gene structures and amino acid sequences were highly similar to rice MADS homologs (77–92%). Most importantly, all of the predicted proteins contain M, I, K, and C domains, definitive of type II MADS (Shih et al., 2014). When the whole genome of moso bamboo was published (Peng et al., 2013), 34 MADS genes were identified (Peng et al., 2013; Cheng et al., 2017). However, the protein lengths and exon numbers were unlike the other Poaceae MADS. Five genes did not have the M domain (PheMADS56-4, PheMADS21, PheMADS14, PheMADS29, and PheMADS90; Cheng et al., 2017), while others were very short and contained only the M domain (PheMADS1, PheMADS5, PheMADS64, PheMADS65; Cheng et al., 2017). Thus far it is unknown whether these differences are due to the starting materials (DNA from *in vivo* flowers in Cheng et al., 2017 vs. RNA from *in vitro* flowers in Shih et al., 2014).

Because MADS proteins are transcription factors, they will form complexes that go to the nucleus. However, most fluorescently tagged BeMADS proteins cannot enter the nucleus when expressed in either *Arabidopsis* protoplasts or in bamboo leaves, but can when expressed in lemmas (Shih et al., 2014). This indicated that correct results can only be shown in the correct materials. Therefore, *in vitro* bamboo flowers are very important for investigations into bamboo reproduction.

## Moving forward

Due to the flowering characteristics unique to bamboo (long juvenile phase, mass flowering, and death after flowering), establishment of controllable *in vitro* bamboo flowering is absolutely required to facilitate timely and effective bamboo breeding. While only self-crosses have thus far successfully produced seeds *in vitro* (Nadgauda et al., 1990), advances in technology, new induction protocols, or alternative hybridization strategies can further the realization of this goal. For example, *D. latiflorus* and *B. edulis* plantlets induced to flower *in vitro* were successfully transferred to the greenhouse, where they continued flowering (Zhang and Wang, 2001; Lin C. C. et al., 2003; Lin et al., 2005). Perhaps parental bamboo accessions could be induced to flower *in vitro* and transplanted to the greenhouse for further hybridization with other bamboos that are flowering, whether they were induced *in vitro* or *in vivo*. This transplantion strategy avoids the limitations of *in vitro* hybridization, such as high humidity or low wind- or insect-mediated pollination rates. *In vitro* flowers can also be maintained in tissue culture to preserve those flowering bamboos that cannot survive in the field. Compared with bamboo vegetative tissues, it is easier to establish bamboo reproductive tissues in a tissue culture system (Lin and Chang, 1998).

Furthermore, our study of bamboo flowering indicated that the use of standard model plant material (ex. *Arabidopsis*) gives misleading results for bamboo (Shih et al., 2014). While many bamboo flower-related genes have been identified via genomics, the mechanisms of flowering, the expression of floral genes and proteins, and other functional analyses must be done in bamboo reproductive tissues. Stable and readily available sources of *in vitro* reproductive tissues offer many advantages for further experiments such as genetic transformation.

As the situation stands today, *in vitro* flowering in bamboo is limited to sympodial bamboos, and only *B. edulis* has thus far been investigated systematically (Lin and Chang, 1998; Lin C. C. et al., 2003). This is a challenge, but our research community hopes to apply the knowledge and techniques reviewed above to further develop tissue culture and *in vitro* flowering protocols for monopodial bamboos, especially for moso bamboo, which has a longer juvenile phase and is the most important monopodial bamboo species for the bamboo industry in subtropical and temperate regions. Furthermore, the work outlined above represents the current state from which researchers can refine floral induction protocols to predictably induce fertile *in vitro* bamboo flowers.

## Author contributions

CSL organized and prepared this manuscript. JLY, JJY, CSL, and XPG contributed to the writing.

### Conflict of interest statement

The authors declare that the research was conducted in the absence of any commercial or financial relationships that could be construed as a potential conflict of interest.
